# Advancing forensic psychiatry in Rwanda: lessons learned and challenges

**DOI:** 10.1080/16549716.2026.2627104

**Published:** 2026-03-16

**Authors:** Isabella D’Orta, Ariel Eytan, Alfred Ngirababyeyi, Charles Nkubili, Paul Nkubamugisha Mahoro, Panteleimon Giannakopoulos, Yasser Khazaal

**Affiliations:** a Division of Institutional Measures, Department of Psychiatry, Geneva University Hospitals, Geneva, Switzerland; b Institute of Global Health, University of Geneva, Geneva, Switzerland; c Neuro-Psychiatric Hospital, Caraes Ndera, Kigali, Rwanda; d Centre de Liaison et de Thérapies Intégratives, Aigle, Switzerland; e Department of Psychiatry, Faculty of Medicine, University of Geneva, Geneva, Switzerland; f Addiction Medicine, Lausanne University Hospital and University of Lausanne, Lausanne, Switzerland

**Keywords:** forensic psychiatry, psychiatric training, mental health services, medical education, ethics, Rwanda

## Abstract

**Background:**

Following the 1994 genocide against the Tutsi, Rwanda showed remarkable resilience in rebuilding its institutions, particularly within the health sector. The genocide against Tutsi left lasting emotional and psychological trauma, highlighting the urgent need to prioritize mental health in national recovery efforts.

**Objective:**

The aim of this article is to present the curricula, outcomes and lessons learned from a forensic psychiatry training in Rwanda.

**Methods:**

Since 1997, a partnership between the University of Kigali and Geneva University Hospitals has supported this goal by co-developing training programs and professional capacity building. A focus of this collaboration has been forensic psychiatry, a field that bridges mental health and the justice system.

**Results:**

During this training major aspects of forensic psychiatry have been addressed: expert evaluations and risk assessments, court-ordered treatments and ethical and legal considerations. Trauma sensitivity has been central as well. The importance of forensic psychiatry extends beyond legal cases, contributing to the management of gender-based violence, substance abuse, and post-traumatic stress disorders. These issues remain significant given Rwanda’s history of genocide against Tutsi and its long-lasting psychological effects.

**Conclusion:**

Continuing the training is essential to strengthen the skills of local professionals in managing complex legal and medical cases, ensuring they are confident in their interactions with the justice system and supporting its effective and ethical functioning. It is essential to strengthen these competencies and the collaboration between local authorities and academic institutions.

## Paper Context

- **Main findings**: Expertise in forensic psychiatry is crucial in post-traumatic settings and specific training is required to address the complex mental health and legal needs of affected populations.
- **Added knowledge**: In Rwanda, a forensic psychiatry training program is underway to strengthen professional competencies and develop a specialized national curriculum.
- **Global health impact for policy and action**: The Rwandan experience may serve as a valuable model for other African countries, particularly those with post-conflict or traumatic histories, to guide the development of forensic mental health capacity.

## Background

Rwanda is a small, landlocked country in East-Central Africa with a population of 14 million people. It is a rapidly developing nation, having made significant progress in social, economic and healthcare sectors over the last decades [1]. Recognized for its political stability and development initiatives, Rwanda has become a model of development in Africa. The country has demonstrated a remarkable ability to implement effective health policies, expanding access to universal healthcare and integrating mental health services into its national health system [2].

After the devastating consequences of the 1994 genocide against the Tutsi, Rwanda has displayed a high capacity of resilience. The profound impact of health-related issues has persisted well beyond the cessation of physical violence, with emotional, psychological, and physiological consequences affecting individuals for more than 30 years [3]. Rwanda has made notable steps in promoting human rights and ensuring better access to healthcare for its population, starting from the universal health coverage. The *Mutuelle de Santé* is a community-based health insurance program introduced by the Rwandan government in 1999 as a cornerstone of the national health strategy, aimed at ensuring universal healthcare access for all citizens [4–6]. The Rwanda Social Security Board (RSSB) provides affordable healthcare access to most of the population, especially low-income citizens [5,6].

Following the collapse of the health system in the immediate aftermath of the genocide against Tutsi and throughout the country’s rebuilding efforts, mental health has been increasingly prioritized, with a strong acknowledgment of its vital importance in public health [3,6,7].

However, despite the tremendous efforts put in place during the decades following the genocide against Tutsi, the mental health toll remains significant [8–10]. Prevalence of PTSD reported may vary considerably, but consistently remains higher than non-trauma exposed populations and it is comparable to that of populations exposed to major traumas, like wars or forced migration [9,11]. Munyandamutsa et al. [11] and Ng and Harerimana [10] estimated the prevalence of PTSD in the Rwandan population to be around 25 – 26%. Studies about major depressive disorder show a prevalence of more than 15% [12], whereas substance disorders are considered an issue especially among young people [13,14].

Efforts to improve mental health care have included expanding communities’ services, strengthening the community-based support and reducing the reliance on the large psychiatric hospital of Ndera (Ndera Neuropsychiatric Teaching Hospital). Raising awareness, reduction of stigma against mental health and its integration into broader health policies have been prioritized too. This holistic approach reflects the nation’s commitment to modernize its healthcare system, addressing the well-being of its citizens in a comprehensive manner. To strengthen this approach, the government has launched in 2024 the 4 × 4 reform, aiming to quadruple the current health-care workforce within four years, with emphasis on education and training [15].

Rwanda has a longstanding tradition of collaboration with European countries and international academic institutions to improve education and strengthen the healthcare system.

Consequently, since 1997 a partnership between the University of Kigali and the Geneva University Hospitals has been established, focusing on co-development of training programs tailored to Rwanda’s mental health needs, academic exchanges and training.

A specific component of this collaboration concerns forensic psychiatry, a field that is increasingly relevant in the Rwandan context due to the growing demand for expert evaluation in the intersection between mental health and the justice system.

These needs have already been highlighted in an earlier publication by this group [16], which introduced the topic and explored the specific gaps and requirements for the development of forensic psychiatric services in Rwanda.

The aim of this paper is to provide a report of the curricula and results of training during last years, to assess its impact and lessons learned. Future avenues for the continuation of this program will also be treated.

## Methods

### Assessment of the training needs

The initial phase comprised a set of preparatory activities aimed at informing context-sensitive implementation through a systematic assessment of local needs, service structures and institutional readiness. During a one-week field visit to Rwanda in 2017, AE, and PNM adopted a mixed formative approach combining clinical workshops with qualitative stakeholder consultations [16]. Key informants at decision-making and governmental roles within the health, justice and security sectors were engaged through open-ended and exploratory qualitative interviews. These interviews sought to elicit locally grounded perspectives on mental health service organization, intersectoral coordination, resource constraints and sociocultural considerations. The objective was of identifying barriers and facilitators to feasibility, acceptability and sustainable integration of the initiative within existing systems of care [17,18].

In parallel two clinical workshops were conducted with psychiatrists, psychologists and nurses working in psychiatric services, predominantly from Ndera. These workshops were designed as platforms for bidirectional knowledge exchange and contextual validation. Each workshop began with a brief overview of the initiative by the investigators followed by a case-based discussion led by a Rwandan mental health professional. The sessions concluded with a collective discussion and synthesis aimed at identifying locally relevant clinical priorities, practice constraints and opportunities for adaptation within existing mental health care structures [19,20].

This first phase of investigation highlighted three domains.

Firstly, the legal framework was judged too vague and insufficiently detailed in the Rwandan penal code. Secondly, there was an expressed need for training of medical students and mental health professionals, related to the current lack of specialists.

Thirdly, the need for a forensic psychiatric unit was a recurrent theme. This concern was based on the perception that a dedicated facility is necessary – a building where forensic patients, often regarded as dangerous, can be admitted without posing a risk of threatening other patients. Episodes of forensic patients aggressing other more vulnerable, such as individuals with intellectual disabilities or severe mental disorders are reported. At the same time, the call for a special forensic unit was linked to the necessity to develop a team with a specific expertise in a field too often perceived as neglected.

Since then, a training program has been implemented, based on these three major areas. The training sessions were conducted by AE, AN, ID, and PNM for one week each year.

**Figure 1.**
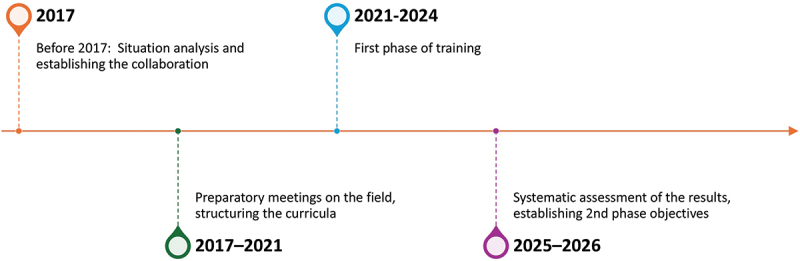
Timeline of the program.

The timeline and phases of the intervention are displayed in Figure 1.

### Overview of the training program

**Figure 2.**
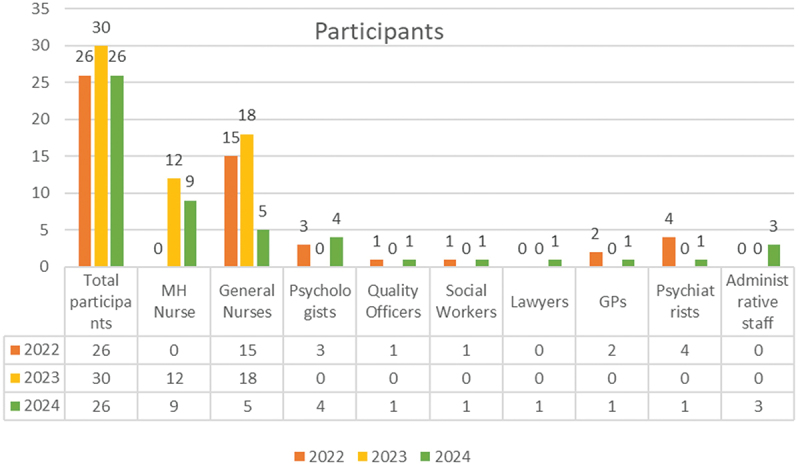
Distribution of participants by profession, per year.

According to the preliminary phases of the identification of needs, the target audience primarily consisted of nurses, medical doctors (including general practitioners and psychiatrists), and psychologists. It also included quality officers, social workers, administrative staff, members of the Health Directorate, and staff from the Rwandan Correctional Service, as shown in Figure 2.

**Figure 3.**
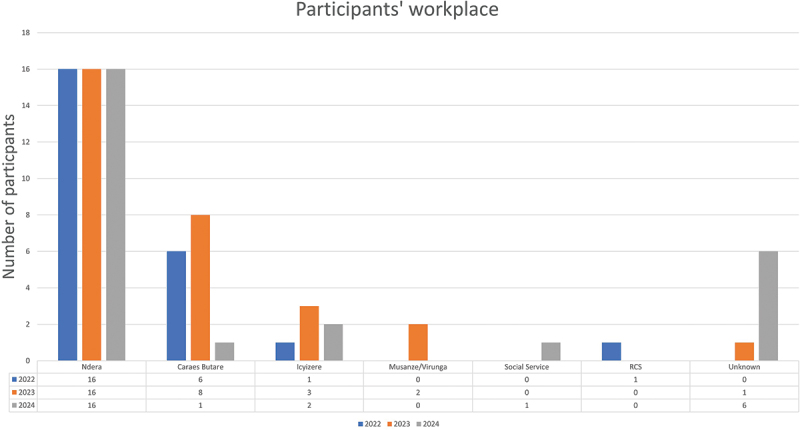
Participants’ workplace.

**Figure 4.**
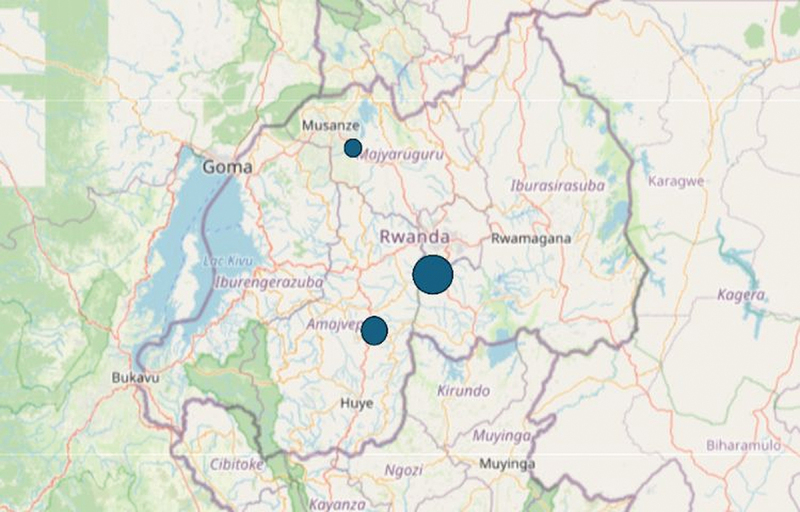
Geographical provenance of participants [21].

The training organizing committee was responsible for arranging some sessions at the Ndera Hospital, while at least two to three days were held in Musanze, a district in the north-west of the country. The aim was to enable the maximum number of mental health staff to participate, at least in part of the event, each year. Participants’ workplace and their geographical provenance are displayed in Figure 3 and in Figure 4.

The program implies at least one full week of training per year and takes place in Rwanda. In addition, after each land-based training, the training is sustained via regular supervisions and interactive workshops conducted remotely, ensuring continuous guidance, knowledge sharing and team coordination across distances.

The five-day program (Table 1) combined lectures, workshops and participant-led case discussions with the aim of strengthening clinical and forensic mental health capacity within the local context. Core content covered areas of general psychiatry, including mood and psychotic disorders. The forensic psychiatry component addressed key practice-oriented themes, such as the preparation of forensic psychiatric reports, court-mandated outpatient care, and mental health needs of detained populations, with particular attention to pathways for accessing care.

**Table 1.** Theorical content of the training program.

General psychiatry, mood disorders and psychotic disorders introduction
Forensic psychiatry introduction
Mental health of detainees
Court-ordered treatment
Writing forensic reports and expertises
Ethical issues

Interactive sessions focused on ethical considerations, policy frameworks and guided case-based discussions, allowing structured reflection on Rwanda-specific legal, cultural and service-delivery contexts. Participant feedback was systematically solicited after each session to enable real-time adaptation of content and ensure contextual relevance.

**Figure 5.**
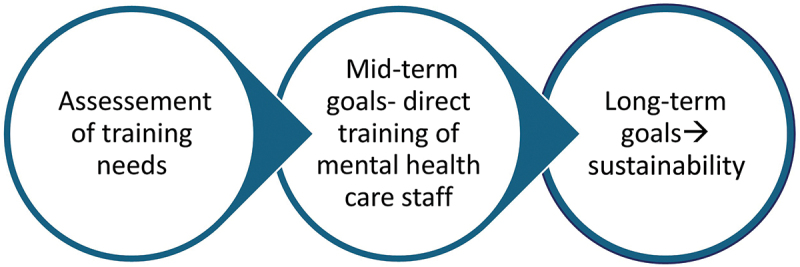
Objectives of the cooperation.

The objectives (Figure 5) were divided into short-, medium and long-term goals. After the initial assessment and launch phase, the medium-term phase – spanning five years – focused on providing comprehensive training in forensic psychiatry to all psychiatrists in the country, as well as offering basic and intermediate training to nurses and other mental health professionals. A third, longer-term phase aimed to develop local trainers through a train-the-trainer approach and to ensure the long-term sustainability of the project.

(1) Each week of the training program included four elements, as follows: **Meetings with relevant stakeholders**, key-informants working at decisional level in the domain of mental health, especially members of the Health Directorate, of the RBC (Rwanda Biomedical Center) and RCS (Rwanda Correctional Services) in order to highlight areas of concern and structure the teaching.
(2) **Briefings with clinical staff** working in different Rwandan public mental health services (mainly in Ndera). The aim of these preliminary sessions was to gather opinions and collect information and perceived needs directly from the field.Moreover, several visits to the wards were conducted, during which clinical cases were presented and discussed.
(3) **Teaching and Conferences**: Based on the inputs that came from the meetings with the stakeholders and the briefing with the clinical staff, these seminars, organized by the Caraes Direction, featured lessons and workshops delivered by the teaching staff on pre-agreed topics.

**Networking opportunities**: Such opportunities were arranged to encourage informal discussions and exchanges after the teaching sessions. These meetings provided a space of dialogue on sensitive and relevant topics, allowing participants to share both personal and collective experience.

A typical training week is displayed in Table 2.

**Table 2.** Typical training week.

Day 1: Activities related to the cooperation, meetings with key stakeholders from government, RBC (Rwandan Biomedical Center), health authorities, and Ndera Hospital leadership.
Day 2: Lectures: introduction to Forensic Psychiatry, discussion followed by clinical case presentation from participants, feedback and workshops with guided discussion. Theorical summary
Day 3: Lectures- General Psychiatry, discussion and link with forensic practice, theorical summary
Day 4: Lecture: Writing forensic reports and expertises, workshops with guided discussion, Lecture: Writing forensic reports and expertises, second part, discussion
Day 5: Interactive workshops on ethical issues in forensic psychiatry, clinical case presentations from residents

To be consistent with the initial objective of enhancing knowledge in forensic psychiatry, each training session began with a brainstorming session involving the participants, followed by the completion of questionnaires designed to identify key topics requiring focused lectures or courses. Notably, from the second session onward, approximately half of the attendees had previously participated in the training, while the remaining participants were attending for the first time. Although some of the topics addressed are well-known in developing countries, particularly in Africa, this analysis focused on their relevance to the Rwandan context, and to assess specific goals and achievements.

## Results

There was a significant need for deeper exploration in forensic psychiatry, given its unique role at the intersection of medicine and law. The results underscored a substantial unmet need for strengthened competencies in forensic psychiatry, a field operating at the interface of healthcare and the legal system. In response, the forensic psychiatry training covered a range of key domains, including:

(1) **Expert Evaluations and Risk Assessments** – This area focuses on assessing individuals’ mental state, competency, and potential dangerousness, often informing legal decisions on criminal responsibility and risk management.The discussion primarily focused on the need to establish a clear intervention setting and organize an expert witness office that would coordinate the activities. Efforts were made to create a standardized draft to follow, in order to make the procedures and reports more consistent, comprehensive and structured. The goal is also to make individual psychiatrists involved in these cases feel less isolated and ensure that they can rely on a solid institutional structure for support.
(2) **Court-Ordered Treatments and Interventions** Forensic psychiatry plays a pivotal role in determining and offering supervision to treatment plans mandated by the legal system. This includes hospitalization, rehabilitation and psychiatric care for offenders with mental disorders.In Rwanda, a number of detainees are placed at Ndera Hospital for evaluation, following a court order. Sometimes, a formal request for a forensic assessment is issued; in other cases, the referral is prompted by behavioral disturbances or the assumption that the individual required pharmacological treatment. The challenges raised by these admissions were addressed in terms of clinical management difficulties and need to reinforce the work force. The staff working with these patients are exposed to a high emotional burden, which can be further exacerbated by the stigma and criticism associated with caring for individuals who have committed offenses. It often leads to feelings of social isolation and in some cases professional burnout.
(3) **Quantitative analysis**: Despite the high burden caused by these patients in terms of clinical and therapeutic effort as well as risk to staff and other patients it emerged from the discussion that they generally represent a heterogeneous and poorly understood population. In particular, they are poorly known from epidemiological, demographic, and clinical perspectives – especially regarding diagnosis, treatment and follow-up. Consequently, the idea arose as a necessity to conduct an in-depth study of a cohort of 100 patients placed from court order in NDERA, through a quantitative analytical statistical approach. The work is currently ongoing after obtaining ethical approval from the IRB of Rwanda University.
(4) **Ethical and Legal Considerations** – The field involves complex ethical dilemmas, such as balancing patients’ rights with public safety, confidentiality issues, and the ethical responsibilities of forensic psychiatrists in legal procedures.Confidentiality is a topic that has been thoroughly discussed. A key issue lies in how to navigate between the obligations and demands of the justice system on one hand and maintain the integrity of the therapeutic role on the other. This balance requires careful consideration of ethical principles, legal obligations, and circumstances of each case.Another concern that was raised several times was the management of agitated patients, and those with chronic conditions, reflecting a growing sensitivity toward the care provided against the patient’s will.In fact, despite the need for a forensic psychiatric unit [16], other aspects were judged more urgent to address. This included the need to improve the management of agitation and situations requiring the use of restraints, the treatment of patients in suicidal crisis, and acute exacerbations of chronic conditions.For most of the participants, the effective management of agitation is perceived as a critical need to ensure the safety and well-being of patients, their families, and healthcare providers. While in certain situations the use of physical or chemical restraints may be necessary to prevent harm, it is unanimously agreed that their use should be minimized and applied only when absolutely necessary. This dialogue highlighted the need to develop a work plan in accordance with established protocols and guidelines, adapted to the local field. Training healthcare professionals in non-invasive de-escalation techniques, as well as in ethical considerations surrounding the use of restraints, is considered essential for improving outcomes and protect both patients and mental health professionals.
(5) **Sensitivity to traumatic issues**: Another aspect to take into account is that, given the country’s troubled and painful past, particular attention should be paid to trauma-related issues, both in patients and in collaborators.Studies have shown how traumatic exposure and PTSD symptoms may shape the attitude towards justice and reconciliation initiative [22,23]. Therefore, developing trauma sensitivity is central as it promotes a mindful and approach towards both patients and collaborators.Since working with forensic issues – often marked by trauma, violence, and similar experiences – can intensify distress and make care provision even more challenging, developing trauma sensitivity becomes even more important

Alongside the more strictly forensic issues, the following topics have emerged as central to the participants of the training, especially those working in the Hospital of Ndera, carrying significant clinical implications.

1. Improving the therapeutic alliance to enhance treatment adherence, mainly for chronic conditions. Poor adhesion to treatment was highlighted, with an emphasis on the need to develop strategies and communication approaches to address this issue.
2. The need for reducing stigma and improving mental health literacy, especially among families and networks of admitted patients. This included the need for increased collaboration with families of patients.The importance of education in the general population was emphasized. A high risk of abandonment and rejection by families was perceived following the diagnosis of a mental health disorder or even after a single consultation, leading to disastrous consequences for the care process. In the Rwandan society, families play a crucial role in the management of psychiatric patients, and their cooperation is considered essential. To address this issue, the participants emphasized the need for improved communication and the ability to provide information on psychopathology in a clear and accurate manner.

### Quality of training

Finally, two key areas emerged through stakeholder exchanges as priorities for strengthening the overall quality and reach of training:

1. Sustaining continuing professional development through the expansion of distance-based training programs and other innovative learning modalities, with the aim of increasing accessibility and participation across services.
2. Developing bilingual training programs (English and French) to ensure equitable access across generations of mental health professionals. Participants highlighted that younger professionals in Rwanda are more likely to be proficient in English, whereas older practitioners predominantly use French. Following the 1994 genocide against Tutsi, English was progressively adopted as the primary language of instruction and administration, reflecting Rwanda’s engagement with international partners, economic modernization and regional integration – particularly within the East African Community [24,25].

## Discussion

The aim of this paper is to describe the strengths and limitations involved in developing a training program in a specific area of mental health – forensic psychiatry – in an African country. The primary objective is to document and analyze the deployment of a training framework as a case study, with the aim of informing the adaptation and scale-up of similar programs in other settings and neighboring countries in Africa facing persistent shortages of mental health professionals and trainers.

The main objective is to offer a framework for similar programs developing in other settings or neighboring countries in Africa, where there is a shortage of mental health professionals and trainers [2,8,26,27].

A secondary aim is to start an interim evaluation of this long-term project that has been ongoing for several years in various forms, with personnel rotating both within the visiting team and the local Rwandan team. An interim evaluation allows the assessment of resources, in-depth post-training evaluation and adaptation of future sessions based on the results. It also contributes to reinforce local and international partnership guaranteeing continuity and sustainability of the training program.

## Pedagogy

While there is increasing commitment to building the local mental health workforce in Africa, the consensus on the most effective strategies for training health care workers and for evaluating the impact of such training efforts is still limited [28].

Training programs in LMICs are expected to prepare professionals who can easily assume roles in service provision and, at the same time, contribute to the stabilization of the workforce. As shown by Liu [28] in this way it becomes possible to build a robust mental health workforce.

Several points and reflections have been considered to guide the construction of the forensic didactic program in Rwanda. From a general and theorical pedagogical perspective, problem-based learning (PBL) is an interactive and learner-centered instructional model that is well accepted and widely implemented in undergraduate medical education. However, the use of PBL in most postgraduate and continuing medical education programs remains limited and studies evaluating its effectiveness are still relatively scarce [29]. On the other hand, traditional didactic lectures (TDL) continue to be a common teaching approach in postgraduate and continuing medical education. Nevertheless, this model often fails to assess learners’ self-efficacy, to identify their educational needs and encourage the use of shared decision-making, which is an important part of modern medical practice [28,30].

From a practical point of view, two central aspects were considered. First, there was the need to provide a participant-centered, interactive environment to improve critical thinking, team collaboration and clinical reasoning. On the other hand, the participants in this program specifically requested to begin each session with a general review of core psychiatry topics – in other words, they asked for lectures (most requested topics were mood disorders and psychotic disorders).

For this reason, a blended program was organized. It begins with an initial section of theoretical lectures as requested by the participants, followed by a more practical part, with problem-based learning, clinical cases and the discussion of key topics.

Finally, we opted for an approach that integrates PBL with traditional didactic lectures (so-called *blended approach*). Brief focused post-PBL lectures allow learners to first engage in real-world case discussions and then consolidate their theoretical knowledge. Final lectures also allow any gaps that remain after the practical sessions to be addressed [29].

### Practical experiences

The program was iteratively shaped through the ongoing integration of participant feedback. Rather than relying on formalized assessment tools, facilitators systematically solicited participants’ reflections at the conclusion of each session. This adaptive, feedback-informed approach enabled real-time adjustments to content and delivery, enhancing contextual relevance and perceived effectiveness.

Participants reported that overviews of general psychiatry topics (mood disorders, psychotic disorders, fundamental diagnostic principles) helped them feel more prepared to engage with the complex material that followed.

Working through clinical and forensic case scenarios, learners practiced differential diagnoses, assessing risk and discussing management options. These exercises encouraged active participation and facilitated the exchange of perspectives.

An important element that emerged spontaneously was the need for protected space to reflect on issues specific to Rwandan society and the local mental health system. Participants were given time to discuss these themes privately in Kinyarwanda, without pressure to share personal reflections with external faculty.

This structure had to be flexible enough and adaptable to the limited training time available.

## Cultural sensitivity

In mental health, it is essential to prioritize educational and training programs that not only advance theorical and scientific knowledge but also ensure its adaptation to local cultural realities.

Assessment of cultural appropriateness concerns both therapeutic interventions and teaching activities. As most psychological interventions are developed and validated in Western contexts, the need for cultural adaptation is widely recognized as essential [31–33]. Cultural adaptation involves modifying an intervention protocol to take into account the patient’s language, the cultural background and contextual factors [31,33]. Symptoms manifestations may vary across cultures: for instance depression may present differently in non-Western contexts [32,33], altering clinical expression and help-seeking patterns [32,33].

In this sense, culturally adaptation becomes especially important when a teaching intervention is delivered by Western-based psychiatrists in non-Western settings, such as low- and middle-income countries [32,33].

Cultural appropriateness of the didactic intervention has been considered a central aspect. In this regard, the team that designed and organized the project was mixed, including permanent members of Rwandan origin with in-depth knowledge of the field and of the Rwandan mental health system.

Their continuous involvement guaranteed the cultural relevance of the intervention and its sustainability and long-term integration within the local mental health system.

Moreover, the presence of Rwandan members reinforced the co-construction process and the legitimacy of the program. They facilitated connections with relevant stakeholders, supported the dialogue between Western-based instructors and local practitioners and provided information on local clinical approaches and expectations.

The support and involvement of high-level local stakeholders – such as the Director of Ndera Hospital – significantly increased the program’s acceptance among mental health professionals in Rwanda.

## Trauma related issues and training in post- conflicts regions

One challenge we encountered was adapting our knowledge on topics such as forensic psychiatry – typically grounded in a Western context – to post-conflict settings, as in the case of post-genocide Rwanda.

If capacity building is fundamental to develop mental health care that functions efficiently, it is also essential to consider the specificities linked to training in post-conflict settings [34]. It should be recalled that survivors of the genocide against Tutsi in Rwanda were exposed to extreme levels of physical and psychological violence [10]. Studies have reported that 94% of people in Rwanda during the genocide against Tutsi experienced at least one genocide-related event, including witnessing the murder of family members, having their property and homes destroyed, or having their lives threatened [23]. As a result of these experiences, traumatic stress symptoms remain highly prevalent. Moreover, the high burden related to displacement and migration before and after the genocide against Tutsi adds another layer of vulnerability, particularly for women. Prolonged exposure to trauma, loss of social support and language barriers may further exacerbate these issues [10].

The most used training approaches in conflict settings in Africa include mhGAP [35] task-shifting/task-sharing, and Mental Health and Psychosocial Support Services (MHPSS) [36] as well as counsellor training and psychotherapist training [37,38]. While there are relatively few high-quality RCTs in African post-conflict settings – and considerable heterogeneity in outcome measures and follow-up periods [39], the available evidence identifies several elements to ensuring sustainability [36,39,40]. These include strong supervision mechanisms and meaningful community engagement. Capacity building must be understood as a process, not an event, with regular supervision and ongoing training remaining critical for maintaining program effectiveness and long-term viability [37].

For this reason, during the program great importance was given to creating space for collective reflection on topics related to forensic psychiatry and the specific characteristics of the country. Although theoretical aspects drawn from the European/Swiss background were used as examples (such as policies or the application of criminal or civil codes), group reflection was actively encouraged to develop a clinical practice suited to the local context and to promote genuine ownership among the staff. Within this context the central role of the family emerged as a great strength in Rwandan society and a fundamental pillar for healing.

## Collaboration between LMICs (low- and middle- income countries) and HICs (high- income countries)

There are several models of collaboration between LMICs and HICs – including project-based partnerships, grant-linked initiatives, collaborations involving the public sector, NGOs or faith-based organizations [37].

It is therefore important to carefully consider which model of collaboration is most appropriate in each context. The literature on global health partnerships shows that equitable and sustainable collaboration requires mutual respect, shared decision-making, and long-term commitment, rather than short-term or top-down interventions [41,42].

A factor that must be considered is the so-called ‘allure of the West,’ which can lead trained professionals to migrate, contributing to brain drain. Training provided by foreign professionals is therefore a double-edged sword: while it helps build local capacity, it must be complemented by incentives that support retention within the country [37].

## Strengthening research

Finally, one way to help reinforce health systems is by improving health research capacity, to facilitate the development of a local evidence base for health interventions to support a stronger healthcare workforce and build the capacity to rigorously evaluate programs [28,34,43,44]. In this perspective, the project to analyze quantitative data from Ndera Hospital is considered an important step in this direction, not only to obtain direct and reliable information, but also to strengthen local capacity for data collection, interpretation, and evidence‐informed decision-making

## Next steps

As previously underlined, Rwanda is advancing community-based mental health care by incorporating services into primary healthcare settings, training general healthcare workers, and implementing outreach programs to increase awareness and reduce stigma [45]. The country is also expanding access to specialized care through district hospitals and fostering international collaborations to enhance mental health policies and resources [11].

Forensic psychiatric expertise is meaningful not only to answer to specific legal questions, but also to manage properly cases involving gender-based violence, substance abuse and post-traumatic stress disorders. These issues remain significant given Rwanda’s history of genocide and its long-lasting psychological effects.

Continuing the training is essential to strengthen the skills of local professionals in managing complex legal and medical cases, ensuring they are confident in their interactions with the justice system and supporting its effective and ethical functioning. It is essential to strengthen these competencies and the collaboration between local authorities and academic institutions.

New avenues may focus on culturally sensitive care: addressing how norms, language and family involvement influence ethical decision-making. They may also include bringing together local traditions and international standards and discussing the ethical tensions between global best practices and context-specific realities.

Next steps should include the development of specialized curricula focused on forensic psychiatry, legal procedures, and ethical considerations, with the provision of practical training through workshops and case simulations. Allowing the specialization of local experts who can continue to make the project sustainable will ensure the long-term sustainability and continuity of the project.

The final aim will be to establish a sustainable and autonomous forensic training program.

## Data Availability

The data that support the findings of this study are available from the corresponding author upon reasonable request.
